# Analysis and experimental validation of IL-17 pathway and key genes as central roles associated with inflammation in hepatic ischemia–reperfusion injury

**DOI:** 10.1038/s41598-024-57139-2

**Published:** 2024-03-18

**Authors:** Siyou Tan, Xiang Lu, Wenyan Chen, Bingbing Pan, Gaoyin Kong, Lai Wei

**Affiliations:** 1 Department of Anesthesiology, Hunan Provincial People’s Hospital (The First Affiliated Hospital of Hunan Normal University), Jiefang West Road NO. 61, Changsha, 410005 China; 2 Clinical Research Center for Anesthesiology of ERAS in Hunan Province, Changsha, China

**Keywords:** Hepatic ischemia–reperfusion injury, Inflammation, IL-17, Bioinformatics, Cytokines, Chemokines, Biomarkers, Experimental models of disease, Preclinical research

## Abstract

Hepatic ischemia–reperfusion injury (HIRI) elicits an immune-inflammatory response that may result in hepatocyte necrosis and apoptosis, ultimately culminating in postoperative hepatic dysfunction and hepatic failure. The precise mechanisms governing the pathophysiology of HIRI remain incompletely understood, necessitating further investigation into key molecules and pathways implicated in disease progression to guide drug discovery and potential therapeutic interventions. Gene microarray data was downloaded from the GEO expression profile database. Integrated bioinformatic analyses were performed to identify HIRI signature genes, which were subsequently validated for expression levels and diagnostic efficacy. Finally, the gene expression was verified in an experimental HIRI model and the effect of anti-IL17A antibody intervention in three time points (including pre-ischemic, post-ischemic, and at 1 h of reperfusion) on HIRI and the expression of these genes was investigated. Bioinformatic analyses of the screened characterized genes revealed that inflammation, immune response, and cell death modulation were significantly associated with HIRI pathophysiology. CCL2, BTG2, GADD45A, FOS, CXCL10, TNFRSF12A, and IL-17 pathway were identified as key components involved in the HIRI. Serum and liver IL-17A expression were significantly upregulated during the initial phase of HIRI. Pretreatment with anti-IL-17A antibody effectively alleviated the damage of liver tissue, suppressed inflammatory factors, and serum transaminase levels, and downregulated the mRNA expression of CCL2, GADD45A, FOS, CXCL10, and TNFRSF12A. Injection of anti-IL17A antibody after ischemia and at 1 h of reperfusion failed to demonstrate anti-inflammatory and attenuating HIRI benefits relative to earlier intervention. Our study reveals that the IL-17 pathway and related genes may be involved in the proinflammatory mechanism of HIRI, which may provide a new perspective and theoretical basis for the prevention and treatment of HIRI.

## Introduction

Hepatic ischemia–reperfusion injury (HIRI) is prevalent in lobectomy and liver transplantation. The Pringle technique reduces intraoperative hepatic trauma bleeding, and HIRI is induced when blood flow is recanalized^1^. During the initial ischemic phase, hepatocyte necrosis is caused by blockage of blood flow into the liver, resulting in hepatic tissue hypoxia, impaired oxidative phosphorylation, and immune activation^2^. As the reperfusion process begins, large amounts of damage-associated molecular patterns (DAMPs) released by necrotic cells activate pattern recognition receptors (PRRs) on immune cells to mediate a downstream cytokine storm, leading to hepatocyte death, impairing liver function, and affecting prognosis^2,3^.

Identifying the core molecules and mechanisms involved in the pathophysiology of HIRI is crucial and can provide valuable information for early clinical intervention and treatment of HIRI. Most traditional analyses of sequencing data have focused only on differences in genes at the expression level, ignoring the intrinsic causality and association of the macroscopic transcriptional landscape with the set outcome. These analytical approaches that fail to consider the intrinsic linkage may miss some critical information, such as the identification of genes that characterize diseases. Recent advances in machine learning techniques have allowed us to take into account this intrinsic linkage, helping us to build and validate disease models and thus identify characterized disease molecules. The review of publicly available data and exploration based on machine learning methods aimed at obtaining new information is one of the goals of this study. There is a wide variety of machine learning algorithms, and we have integrated single-model approaches in supervised learning models and integrated learning approaches, including the least absolute shrinkage and selection operator (LASSO) algorithm, random forest (RF), and support vector machines-recursive feature elimination (SVM-RFE) algorithms. Each of the three algorithms has its advantages and compensates one another for the shortcomings of the algorithms. In this study, we combined three machine learning algorithms, weighted gene co-expression network analysis (WGCNA), gene set enrichment analysis (GSEA), protein–protein interaction (PPI) network analysis, and other analytical tools to investigate the characteristic vital molecules and pathways involved in HIRI pathophysiology and validate the role of typical molecules and key pathways mediating HIRI by constructing a rodent HIRI model, which can provide information and foundation for further drug development and clinical diagnosis and treatment.

## Methods and materials

### Data processing and download of the HIRI datasets

GSE117915 (https://www.ncbi.nlm.nih.gov/geo/query/acc.cgi?acc=GSE117915) and GSE24430 (https://www.ncbi.nlm.nih.gov/geo/query/acc.cgi?acc=GSE24430) were downloaded from the Gene Expression Omnibus (GEO, https://www.ncbi.nlm.nih.gov/geo/) database. According to the annotation files from the respective platforms, the probe IDs were converted to gene symbols based on the average value of probe loci. If the probes did not correspond to the gene symbols, they were removed. For further analysis, the microarray data were transformed into log^2^ values. Our merged dataset was formed by integrating them using the Combat algorithm implemented in the R package "sva" and removing batch effects^4^.

### Analysis based on LASSO, RF, and SVM-RFE algorithms

The goal of a general linear regression model is to minimize the sum of squared residuals, i.e. to predict the target variable by fitting a linear equation. In a huge amount of gene expression information, there may be a significant number of independent variables that are weak or redundant predictors of the target variable. We would like to identify genes that may have an important impact on disease, a number that is, in general, greatly reduced relative to the original sequencing-based number. In this case, Lasso regression enables feature selection and model sparsity by introducing L1 Regularization (i.e. the Lasso penalty term), which changes the weights of the smallest elements of the coefficient vector to zero^4,5^. L1 Regularization is a technique commonly used in machine learning and statistical modeling to help the model generalize better to unseen data, avoiding overfitting problems. It avoids the situation where a model performs very well on training data but performs poorly on unseen test data. In our data, the probability of overfitting is higher in the previous process of feature gene identification, screening, and model building due to the relatively limited selection of datasets. By selecting features corresponding to non-zero coefficients, LASSO can screen out the features that have the greatest predictive power for the target variable, thus simplifying the model and improving its generalization ability. In addition, this method is especially suitable for model fitting with too many variables and small sample sizes, so we chose this method for the integrated dataset, especially for the case of small sample sizes of sequencing data. The LASSO algorithm was completed using the glmnet package in R software, and the covTest package was adopted to perform the computation of the p-value corresponding to the parameter estimation. The main steps include data preparation and preprocessing, LASSO modeling, and variable screening (SFig. 1A).

RF is an integrated learning method for classification and regression. RF is based on decision tree construction and improves the performance and stability of the model by combining multiple decision trees^6^. The main idea of RF is to construct multiple decision trees by randomly selecting samples and features and then combining their predictions for classification or regression. We chose this algorithm for three reasons: (i) It performs well mainly on classification models, as evidenced by higher classification accuracy. (ii) It also has advantages when dealing with large datasets. (iii) It is able to measure the relative importance of variables for classification while categorizing. The RF workflow mainly consists of two parts: decision tree construction and voting, as shown in SFig. 1B.

SVM-RFE finds the best variables by culling the feature vectors generated by SVM, which is suitable for situations where there are too many variables and the variable selection needs to be fast and automated^7^. SVM is a boundary-based classification method that can be used to filter the features that are most relevant to the target variable. SVM achieves classification by finding maximally spaced hyperplanes in the feature space, which maximizes the distance between hyperplanes and classification boundaries on the training set. RFE optimizes the model on a subset of features by recursively removing the worst-performing features of the model and re-training the model on the remaining features, ultimately resulting in a subset of features on which the model is optimal. This method can be used for feature selection to remove unnecessary features, thus reducing the risk of overfitting. In addition, it can handle nonlinear problems. Therefore, based on the above advantages, we choose to incorporate SVM-RFE into the machine learning algorithm of this study. The algorithm process is divided into two parts, in the first part the initial model is built using the SVM model, then the most relevant features to the target variables are filtered by adjusting the weights of the variables in the model, and finally, cross-validation is used to select the best combination of features. In the second part, feature selection is performed by RFE to reduce feature redundancy and improve model performance, and finally, a smaller subset of features is obtained (SFig. 1C). For large-scale datasets, Lasso can be computationally expensive because all combinations of features have to be considered. RF, on the other hand, has an advantage in handling large data, but on certain sample sets that are relatively noisy, RF's model tends to fall into overfitting. Therefore, both the LASSO algorithm and SVM-RFE algorithm can make up for the shortcomings of RF to a certain extent, which is why we choose the joint machine learning algorithm.

### WGCNA

In order to investigate gene set expression, the WGCNA method is used. At various stages of the following main phases, multiple gene networks were developed and modularized using the WGCNA R package. The samples were clustered in order to identify outliers. Co-expression networks were then created using automated networks. Hierarchical clustering and dynamic tree-cutting functions were used in the modules. To connect modules with clinical characteristics, module membership (MM) and gene significance (GS) were estimated^4^.

### Differential expression analysis

mRNA expression differences were investigated using the Limma package^8^. Adjusted P-values were examined in GEO to account for false-positive results. R package ggord was used to visualize the threshold mRNA differential expression screen, which was defined as |logFC|> 0.5, P-value < 0.05.

### Functional enrichment analysis and PPI network analysis

Biological Process and Kyoto Encyclopedia of Genes and Genomes (KEGG) analyses were conducted on the g: Profiler database (https://biit.cs.ut.ee/gprofiler/gost), which analyzes functional enrichment and conversion of gene lists to grasp biological characteristics^9,10^. We submitted these genes to g: Profiler with P < 0.05 as a cut-off criterion. The PPI information was visualized using the Search Tool for the Retrieval of Interacting Genes (STRING) database^11^. Then, Cytoscape software was adopted to construct PPI networks^12^.

### Expression and diagnosis significance verification

The expression levels of the key genes in non-HIRI samples and HIRI samples were calculated using the Wilcoxon rank-sum test. We further validated the predictive value of the optimal feature genes using receiver operator characteristics (ROC) curves.

### Animal experiments

### Experimental procedure

Eight-week-old male C57BL/6N mice were obtained from the Center of Experimental Animals at the Hunan Provincial People’s Hospital (Hunan Normal University), Hunan, China. Animals were randomly divided into specific groups depending on the different purposes. Mice were bred and housed in a temperature (20–22 ℃) room with a 12/12 h light/dark cycle. All animals had access to food and water. One-week acclimatization period was applied before experiment employment. The animal experiment was divided into two parts. In the first part, mice were divided into 6 groups, namely, Sham group, Ischemia 1 h group, HIRI 1 h group, HIRI 2 h group, HIRI 4 h group, and HIRI 6 h group, to explore the serum levels of IL-17 and liver tissue IL-17A mRNA expression in mice during the different phases of HIRI (before ischemia, after ischemia, and during reperfusion at 1 h, 2 h, 4 h, and 6 h). In the second part, mice were divided into five groups, namely the Sham group, HIRI group, anti-IL17A (pre) + HIRI group, Ischemia + anti-IL17A group, and HIRI + anti-IL17A (post) group, to investigate the effects of anti-IL-17A antibody intervention on the tissue damage, inflammatory responses, and related gene expression in different stages of HIRI (before ischemia, after ischemia and at 1 h of reperfusion).

In the Sham group, animals were dissected after anesthesia and fixation only, and the hepatic hilum was exposed for 1 h and sutured. In the HIRI group, animals were subjected to partial hepatic ischemia for 60 min, followed by open perfusion of blood. In the anti-IL17A (pre) + HIRI group, preconditioning with 100 µg anti-IL-17A antibody (Anti-mouse IL-17A-InVivo, Clone: 17F3, Selleck) was injected into the retro-orbital space before induction of ischemia. In the ischemia + anti-IL17A group, 100 ug of anti-IL-17A antibody was injected into the retro-orbital space after 1 h of blood flow blockade, followed by 6 h of blood flow reperfusion in the liver. In the HIRI + anti-IL17A (post) group, mice received a retro-orbital injection of 100 ug of anti-IL-17A antibody at 1 h of reperfusion, followed by 5 h of reperfusion.

### Randomization and blinding

Mice were randomly assigned using the random number generator in SPSS software (SPSS Inc., Chicago, IL, USA).

Experimental manipulations in mice such as surgical operations, HIRI model construction, and sample acquisition were not blinded. Testing and evaluation of samples, including assessment of hepatic pathological damage, determination of transaminases, inflammatory mediators, and gene expression were blinded. The evaluators of the experimental results were unaware of the sample grouping.

### 70% HIRI model

The mice had free access to water, fasted for 6 h, and then anesthetized with 3% pentobarbital sodium solution by intraperitoneal injection. After fixation, skin preparation, and disinfection, an incision of approximately 2 cm was made along the median line under the raphe, and the hepatoduodenal ligament of the mice was dissected after exposing the hepatic hilum. The arteries, veins, and bile ducts supplying the middle and left lobes of the liver were isolated and then blocked with a noninvasive arterial clip for 1 h, thereby inducing 70% hepatic ischemia^13,14^. Since the blood flow to the middle and left lobes of the liver accounted for approximately 70% of the total blood flow to the liver, pale changes on the surfaces of the middle and left lobes of the liver due to the blockage of the blood supply were observed when the corresponding veins were clamped with arterial clamps. After releasing the vascular clamps, 0.5 mL of 0.9% NaCl solution was dripped into the abdominal cavity, the abdominal incision was sutured, and the liver reperfusion was initiated^15^. At this time, pallor was observed in the middle and left lobes of the liver due to ischemia. In contrast, the color appearance of the right and caudate lobes of the liver was normal pink and was no different from that of the sham group, which means that the 70% HIRI model was successfully constructed.

### Histopathology and morphometry

Liver sections were stained with hematoxylin and eosin (H&E) as previously described^16^. The left lobe of liver tissue was obtained and fixed in 4% formalin solution for 48 h, followed by gradient ethanol dehydration, immersion wax embedding, sectioning, patching, overnight oven at 62℃, xylene dewaxing, gradient ethanol hydration, 4-μm-thick tissue sections for H&E staining, and the sections were placed under a light microscope to observe the pathological changes of liver tissue in each group of mice and Suzuki’s scale (0–4) was used to score the histopathological damage of the liver^17^.

### Serum transaminase, interleukin, and tumor necrosis factor-α (TNF-α) measurement

Original sources of method description as already published^18^. About 1 mL of blood was obtained after rapid removal of the eyeballs in each mouse. The serum was separated by centrifugation at 2000×*g* at 4 °C for 10 min. Serum levels of alanine transaminase (ALT), aspartate aminotransferase (AST), interleukin- 6 (IL-6), IL-17A, and TNF-α were determined using a Synchron CX7 analyzer (Beckman Coulter, USA) in the Clinical biochemical Laboratory of Hunan Provincial People’s Hospital with a standard biochemical laboratory assay. (Changsha, China).

### RNA isolation and quantitative real-time PCR

Total RNA was isolated from tissues or cells using Trizol (Pufei), and RNA concentration and purity were measured using a spectrophotometer^16^. RNA was reverse transcribed using the PrimeScript RT reagent Kit (Takara) following the manufacturer’s instructions, and quantitative PCR was performed using a CFX96 Real-Time System (Bio-Rad) with SYBR Green Supermix (Bio-Rad) in accordance with the manufacturer’s instructions. All reactions were performed in triplicate, and specificity was monitored using melting curve analysis. See supplemental Table 1 for the primers used.

### Statistical analysis

Sample sizes of animals required for the experiments were estimated using an estimation method based on mean differences. For comparing antibody intervention groups for serological markers and PCR assays for tissue mRNA expression, the sample size for each group was determined to be 10 and 4 mice per group, respectively. For comparing IL-17A protein levels and mRNA expression differences between groups, the number of mice in each group was determined to be 4 mice per group. Data were analyzed, and plots were generated using GraphPad Prism version 10.0 (GraphPad Software, Inc., La Jolla, CA). All values are expressed as means ± SD. For multi-group comparisons, statistical differences between groups were analyzed using one-way ANOVA with Tukey’s post hoc test. Values were considered as statistical differences for P < 0.05.

### Ethics approval and consent to participate

All aspects of the study protocol received full board review and approval by the Ethics Committee for the use of experimental animals in the Hunan Provincial People’s Hospital (Hunan Normal University). (Reference number: 2022-02, date of approval: 04/01/2022).

## Results

### Functional annotation between HIRI and non-HIRI samples

**Figure 1:**
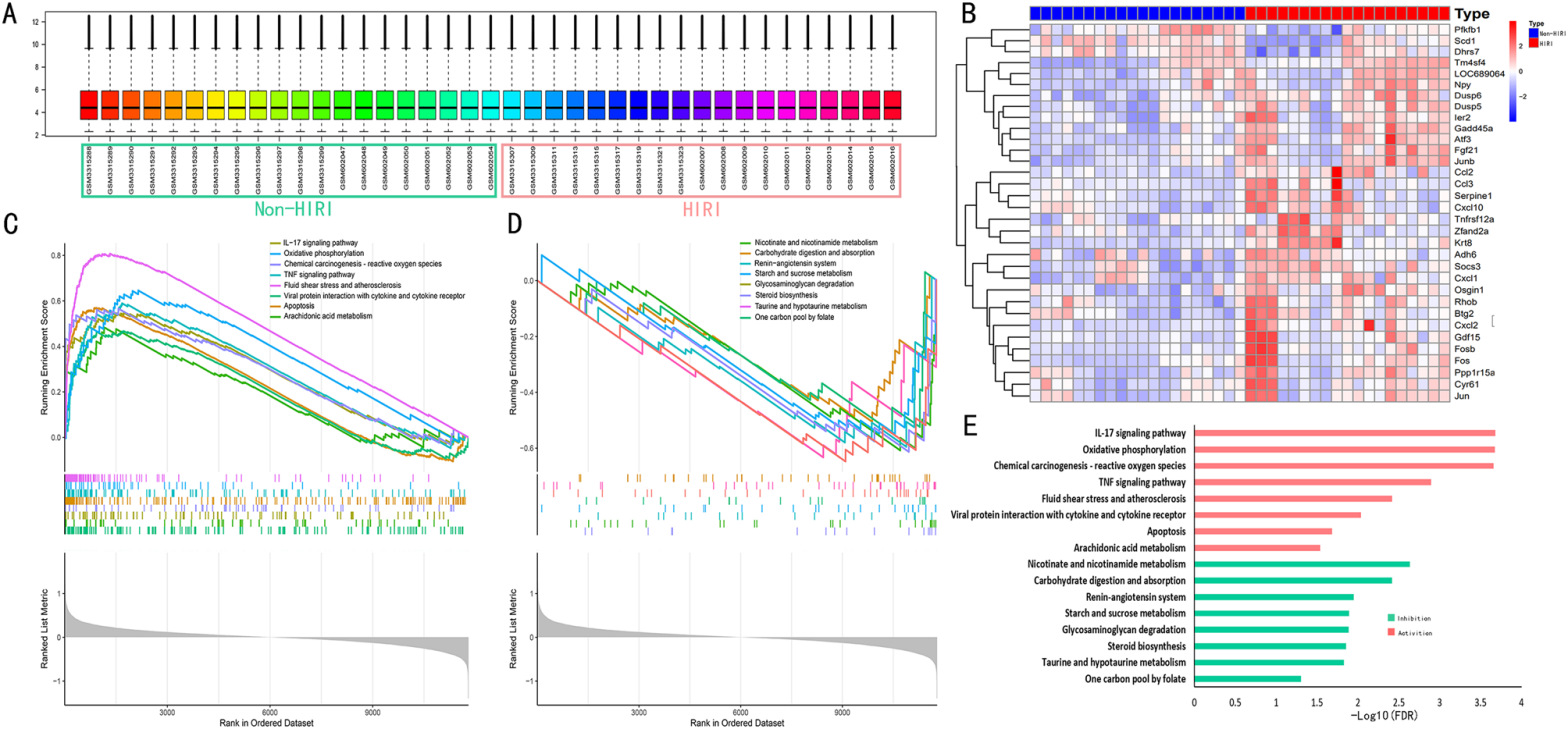
Gene set enrichment analysis. (**A**) Gene expression level statistics of the integrated dataset after removing batch effect. (**B**) The heatmap of HIRI-related gene expression levels. (**C**) The main biological pathways that are significantly enriched in the HIRI group. (**D**) The main biological pathways are significantly enriched in the non-HIRI group. (**E**) The main biological pathways are significantly enriched in the non-HIRI group and HIRI group.

In this study, we merged two microarray datasets from the GEO database, including the GSE117915 and GSE24430 datasets, and obtained 20 control and 19 HIRI samples. Before data analysis, we removed the batch effect from different batches between the datasets (Fig. 1A). As shown in the heat map, the two groups of samples exhibit distinct gene expression profiles (Fig. 1B). To clarify the functional and biological pathways differences between HIRI and control samples, we performed a GSEA analysis of KEGG and screened significantly enriched signaling pathways (Fig. 1C–E). Inflammation-related pathways or biological processes were selected for presentation in Fig. 1E. IL-17 signaling pathway, oxidative phosphorylation, TNF signaling pathway, fluid shear stress and atherosclerosis, viral protein interaction with cytokine and cytokine receptor, and apoptosis were activated, and significantly enriched in HIRI samples (Fig. 1C,E). In contrast, taurine and hypo-turine metabolism, glycosaminoglycan degradation, steroid biosynthesis, and nicotinate and nicotinamide metabolism were enriched in non-HIRI samples (Fig. 1D,E).

### Identification of feature genes by integrating multiple bioinformatic algorithms

**Figure 2:**
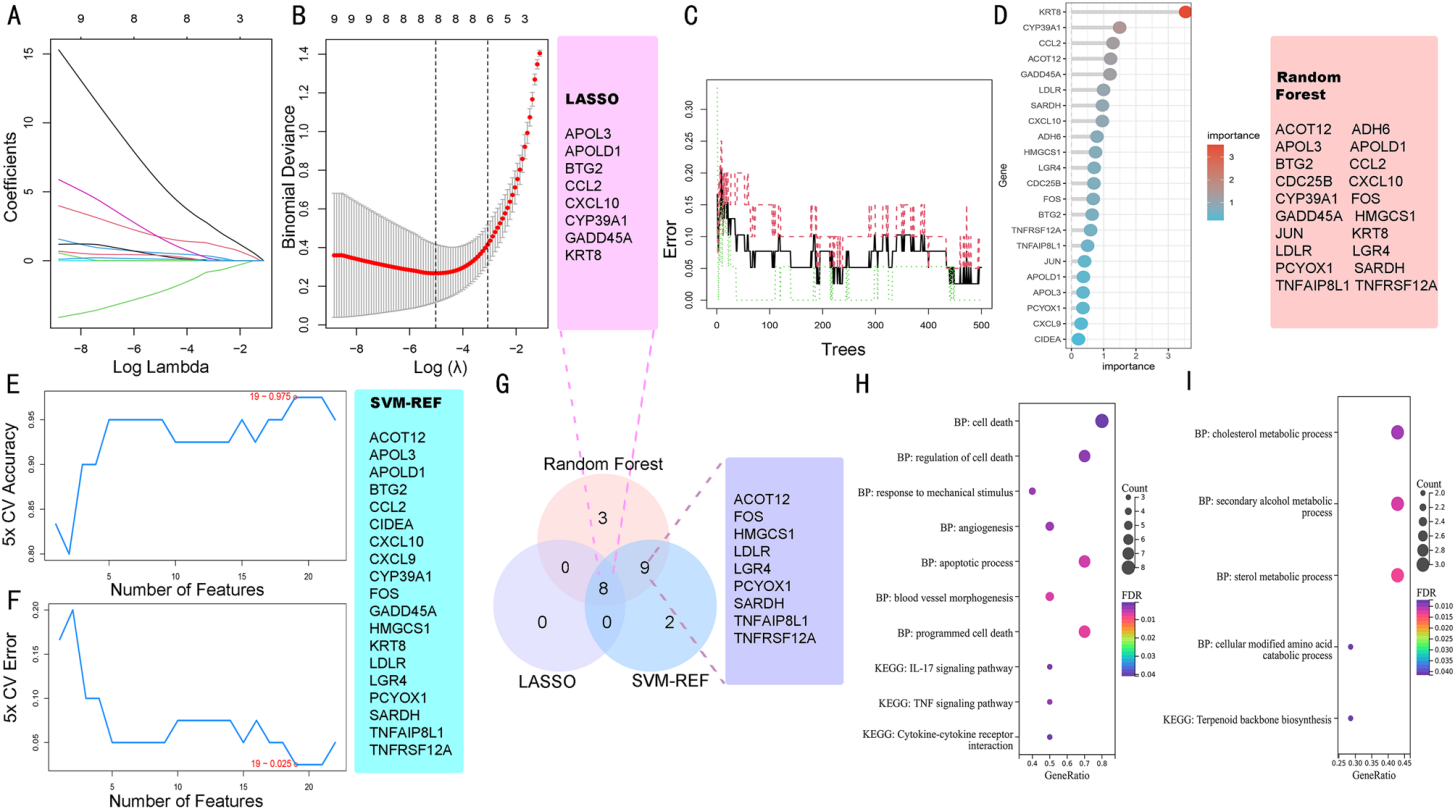
LASSO, RF, and SVM-RFE algorithms were integrated to identify the feature genes. (**A**) LASSO coefficient profiles of the candidate feature genes and the optimal lambda were determined when the partial likelihood deviance reached the minimum value. Each coefficient curve in the left picture represents a single gene. (**B**) The solid vertical lines represent the partial likelihood of deviance, and the number of genes (n = 8) corresponding to the lowest point of the cure is the most suitable for LASSO. (**C**) RF for the relationships between the number of trees and error rate. The x-axis represents the number of decision trees, and the y-axis is the error rate. (**D**) The relative importance of candidate genes is calculated in random forest. (**E**,**F**) The SVM-RFE algorithm was used to further candidate feature genes with the highest accuracy and lowest error obtained in the curves. The x-axis shows the number of feature selections, and the y-axis shows the prediction accuracy or prediction error. (**G**) Venn diagram showing the 17 overlapping feature genes shared by any two algorithms. (**H**) Functional enrichment analysis of the upregulated overlapping genes. (**I**) Functional enrichment analysis of the downregulated overlapping genes.

Three algorithms were employed to identify the putative feature genes. Specifically, we identified eight feature genes as the diagnostic markers for HIRI using the LASSO analysis (Fig. 2A,B). For the RF algorithm, the top 20 feature genes were determined (Fig. 2C,D). Furthermore, using the SVM-RFE algorithm, 19 feature genes were selected after fivefold cross-validation (CV) of the highest accuracy and lowest error (Fig. 2E,F). Finally, the intersection of the feature genes obtained from the above three algorithms was taken, and a total of 17 optimal feature genes were identified, which could be used as potential diagnostic markers for HIRI and may be critical genes involved in HIRI progression (Fig. 2G, supplemental Table 2). Functional enrichment analysis indicated that these feature genes upregulated were significantly associated with regulating cell death, apoptosis, and IL-17 signaling pathway (Fig. 2H). The downregulated feature genes are mainly enriched in the metabolic process (Fig. 2I).

### WGCNA and screening of hub modules

**Figure 3:**
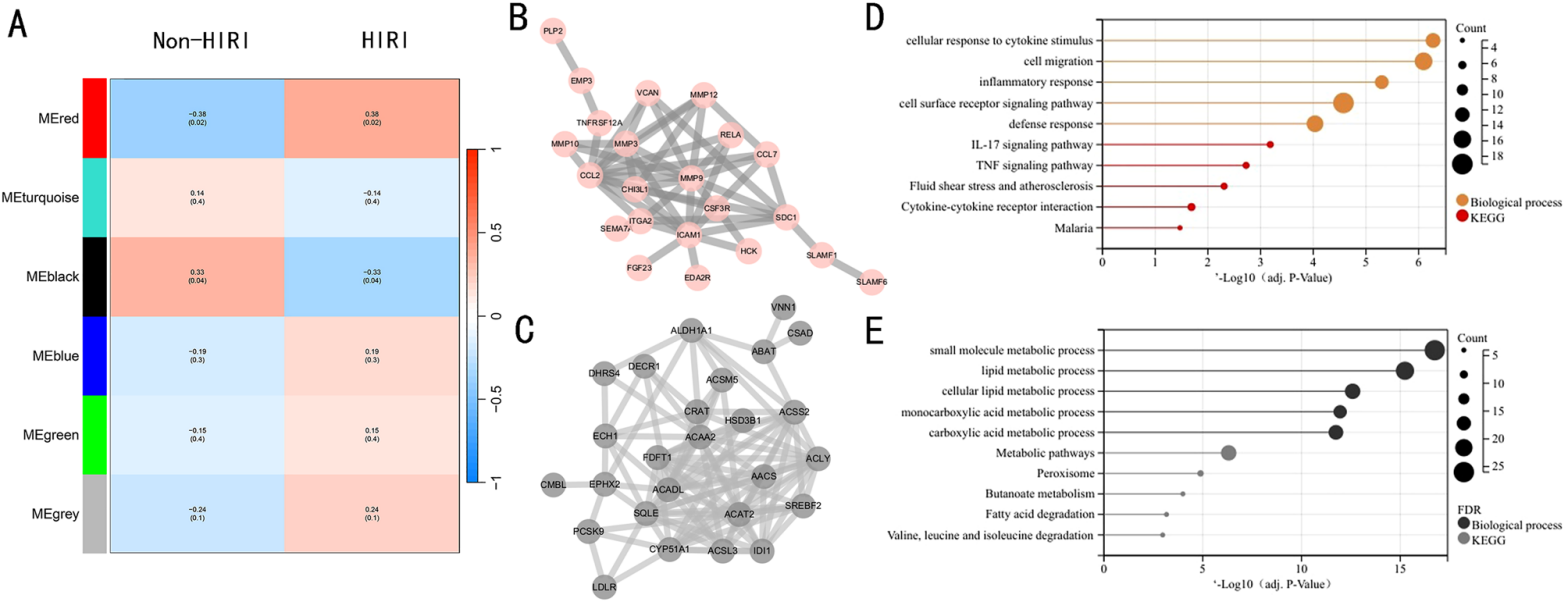
Weighted gene co-expression network analysis. (**A**) Heat map of module–trait correlations. Red represents positive correlations, and blue represents negative correlations. (**B**) The PPI network of the red module. (**C**) The PPI network of the black module. (**D**) Functional annotation of the red module. (**E**) Functional annotation of the black module.

20 control samples and 19 HIRI samples were preferred to cluster the samples and exclude the obviously aberrant samples by setting a threshold, as shown in SFig. 2A. Then, as shown in SFig. 2B, we set the soft threshold to 10 when R^2^ > 0.9 and the average connectivity is relatively high (SFig. 2B,C). 6 modules were identified for further study after merging the strongly associated modules using a 0.18 clustering height limit. The primed and merged modules were displayed under the clustering tree (SFig. 2D). The correlation between modules was examined next (SFig. 2E), and the reliability of module delineation was demonstrated by transcription correlation analysis within modules (SFig. 2F). The frontal correlations between ME values and clinical features were used to investigate the link between modules and clinical symptoms. The red module positively correlated with HIRI (r = 0.38, P = 0.02) and negatively linked with control samples (r = – 0.38, P = 0.02). In contrast, the black module was negatively connected with HIRI (r = – 0.33, P = 0.04) and positively correlated with control (r = 0.33, p = 0.04) (Fig. 3A). PPI network analysis of the red module (Fig. 3B) and the black module (Fig. 3C) revealed that both the genesets are richly interconnected, suggesting that the two modules are both recognized as an interconnected whole in the WGCNA approach (supplemental Table 3). Biological process and KEGG analysis revealed that genes in the red module were significantly enriched in cellular response to cytokine stimulus, cell migration, inflammatory response, defense response, and IL-17 signaling pathway (Fig. 3D). For the black module, genes were mainly associated with the small molecule metabolic process, lipid metabolic process, cellular lipid metabolic process, and metabolic pathways (Fig. 3E).

### Identification of the potential genes, functional enrichment analysis, PPI network analysis, and assessment of the expression and diagnosis significance

**Figure 4:**
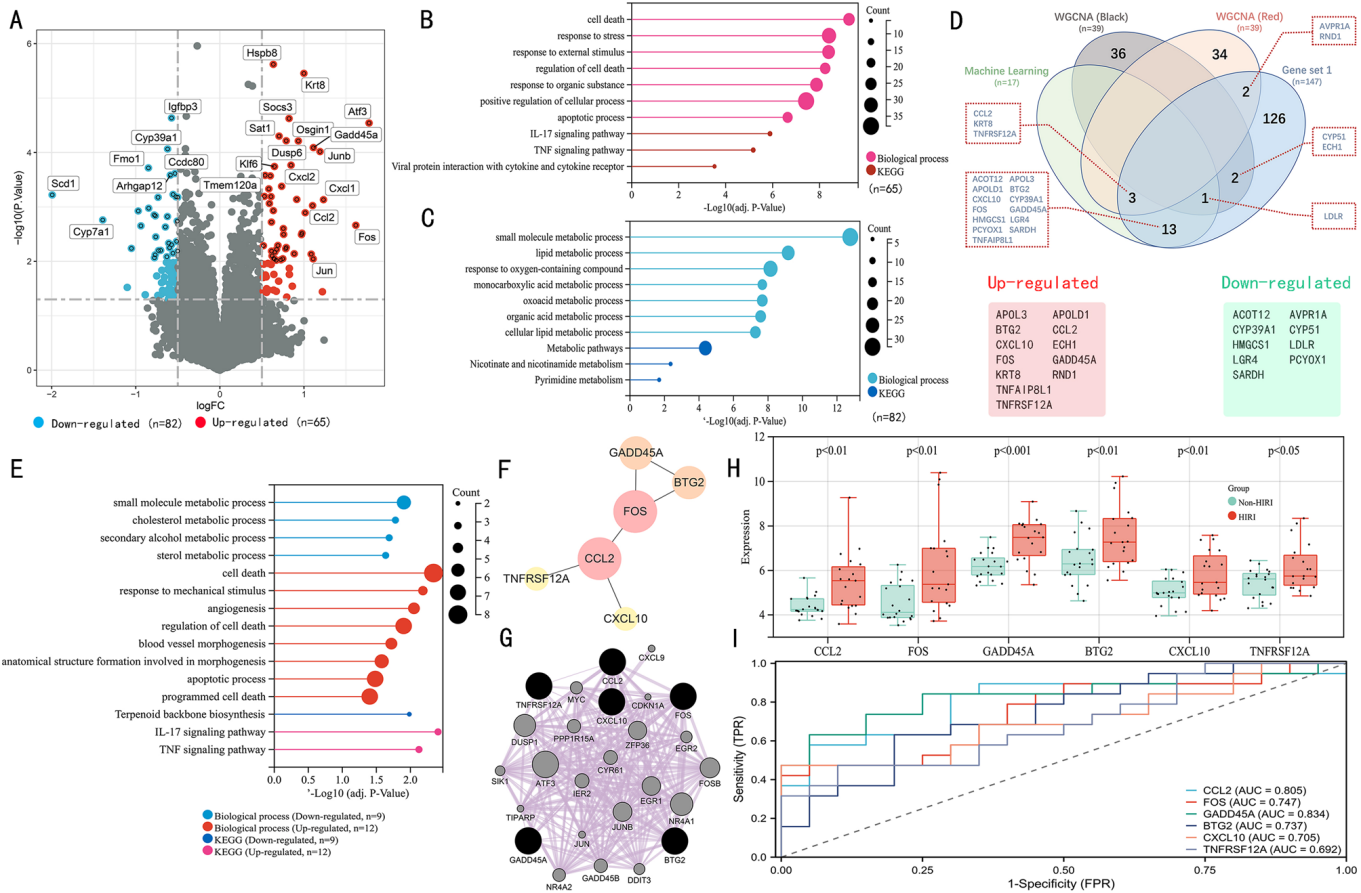
Identification of the potential genes in HIRI. (**A**) The volcano plot of HIRI-related DEG expression (Filter threshold: P < 0.05 AND |logFC|> 0.5). (**B**,**C**) Biological process and KEGG analysis of DEGs. (**D**) Venn diagram summarizing machine learning algorithms, WGCNA, and DEGs. (**E**) Functional enrichment analysis of 21 overlapping genes. (**F**) The PPI network of the potential genes, based on the CytoHubba algorithm, visualizes the importance of genes according to the node's size. (**G**) GeneMANIA analyzed the potential genes and their co-expression genes. (**H**) Verification of expression level of the potential genes in HIRI. (**I**) Estimating the diagnostic performance of the potential genes.

**Figure 5:**
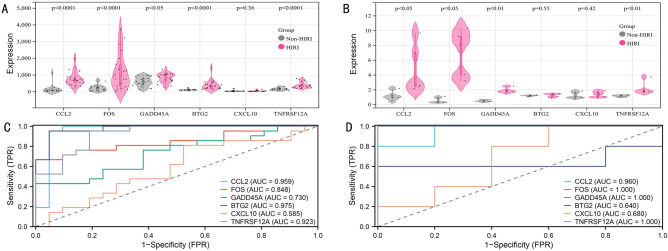
External validation of potential gene expression and diagnostic efficacy. (**A**) Expression levels of potential genes were verified using the GSE12720 dataset. (**B**) Validation of expression levels of potential genes using the GSE14951 dataset. (**C**) Validation of diagnostic efficacy of potential genes using the GSE12720 dataset. (**D**) Validation of diagnostic efficacy of potential genes using the GSE14951 dataset.

Using P < 0.05 and |logFC|> 0.5 as screening criteria, 147 differentially expressed genes (DEGs), including 82 downregulated and 65 upregulated genes, were identified (Fig. 4A, supplemental Table 4). The 65 upregulated genes were notably enriched in cell death, response to stress, response to external stimulus, apoptotic process, and IL-17 signaling pathway (Fig. 4B). In comparison, the 82 downregulated genes were mainly associated with small molecule metabolic process, lipid metabolic process, response to oxygen-containing compound, and other metabolic pathways (Fig. 4C). To further identify the optimal genes driving the pathophysiology of HIRI, we took the intersection of three gene sets from machine learning algorithm (n = 17), WGCNA (red module = 39; black module = 39), and DEGs (gene set 1, n = 147) to obtain a total of 21 genes, including 12 upregulated genes and nine downregulated genes (Fig. 4D). Among the 21 genes, BTG2, CCL2, CXCL10, FOS, GADD45A, KRT8, TNFAIP8L1, and TNFRSF12A were enriched in the biological process of cell death; CCL2, CXCL10, and FOS were associated with IL-17 pathway; ACOT12, AVPR1A, CYP39A1, HMGCS1, LDLR, and SARDH related to small molecule metabolic process (Fig. 4E). By performing PPI network analysis of these 21 genes, 6 genes were identified as potential hub genes while having the highest importance based on the CytoHubba algorithm, namely: BTG2, CCL2, CXCL10, FOS, GADD45A, TNFRSF12A (Fig. 4F). Co-expression gene prediction of the 6 genes by GeneMANIA plug-in revealed that these genes were mainly associated with positive regulation of programmed cell death and cellular response to stress (Fig. 4G). We further validated the expression levels of the 6 genes in 19 HIRI samples and 20 control samples. The expression levels of the genes were significantly upregulated in the HIRI samples, indicating their potential roles during the progression of HIRI (Fig. 4H). Besides, to quantitatively assess the diagnostic and predictive value of the optimal genes, we conducted an ROC curve analysis (Fig. 4I). The area under the curve (AUC) values of ROC curves were GADD45A of 0.834, CCL2 of 0.805, FOS of 0.747, BTG2 of 0.737, CXCL10 of 0.705, TNFRSF12A of 0.692, demonstrating that these potential genes enabled to estimate the progression and had a high diagnostic value for HIRI. Meanwhile, GSE12720 (including liver biopsy samples from 21 liver transplant recipients before transplantation and 1 h of reperfusion) and GSE14951 (including liver biopsy samples from 5 pre-transplantation and 5 2-h of reperfusion) were used for external validation of the potential genes. CCL2, GADD45A, FOS, and TNFRSF12A were significantly upregulated in both datasets (Fig. 5A,B) and showed efficient diagnostic efficacy for HIRI (Fig. 5C,D).

### Liver and blood IL-17A were significantly elevated in the early stages of HIRI

**Figure 6:**
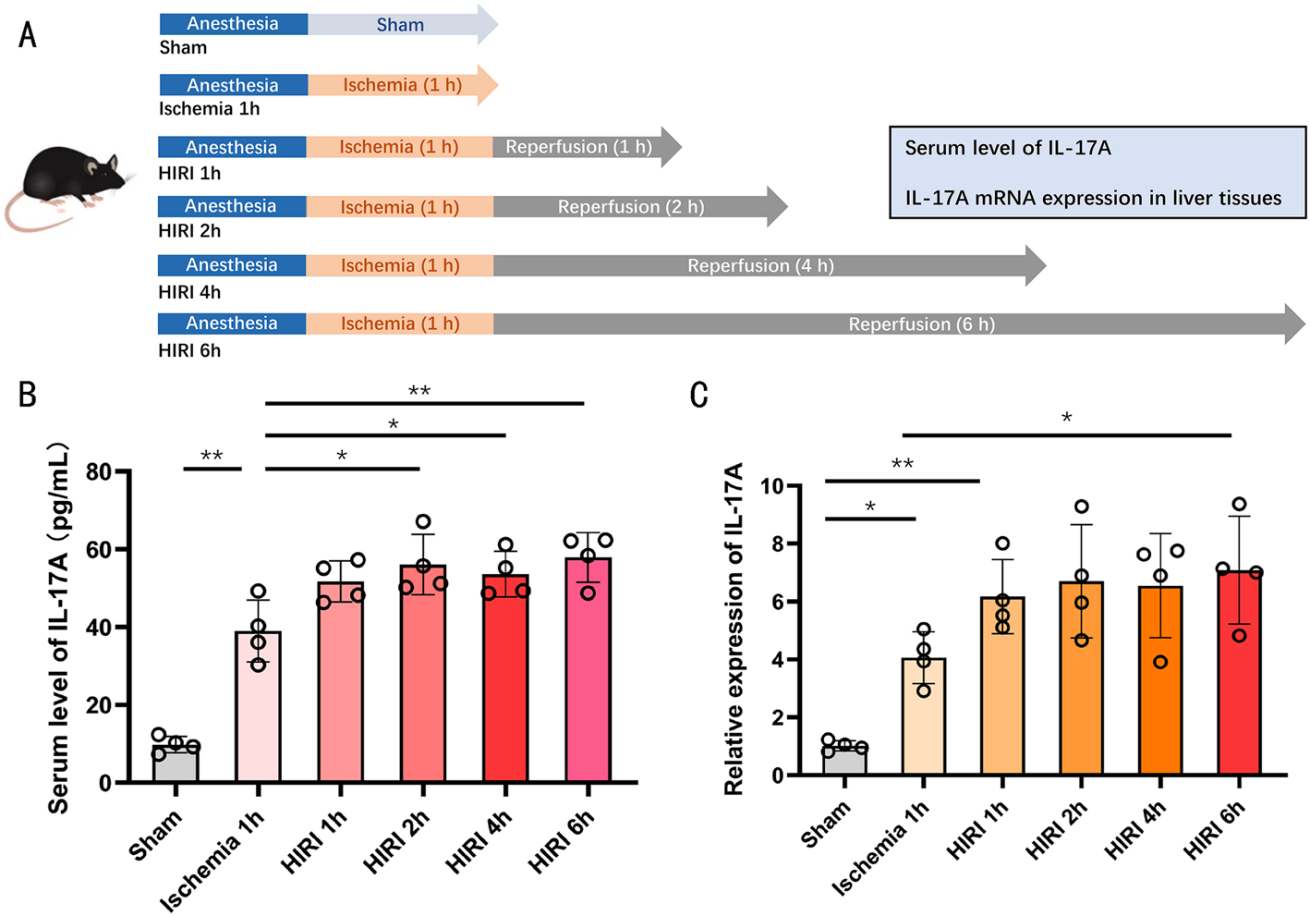
Serum levels and tissue expression of IL-17A in different stages of HIRI. (**A**) Pattern diagram of the experiment. (**B**) Serum levels of IL-17A at the ischemic stage of HIRI and 1 h, 2 h, 4 h, and 6 h of liver reperfusion (n = 4, each group). (**C**) Expression of IL-17A mRNA in liver tissues during the ischemic phase of HIRI and at 1 h, 2 h, 4 h, and 6 h of liver reperfusion (n = 4, each group). *P < 0.05 and **P < 0.01. All data are representative of 3 replicate experiments.

None of the animals and samples included in the study were invalidated or excluded. The experimental procedure is shown in Fig. 6A. We found that IL-17A levels in serum appeared to be elevated during the ischemic phase, and serum IL-17A levels were significantly increased in the ischemic group as well as in all HIRI groups compared with the sham group (Fig. 6B). After 1 h of restoration of blood flow to the liver, IL-17A levels in serum were elevated, indicating an acceleration of the process of reperfusion injury, which reached a peak at 2 h. Subsequently, IL-17A levels in serum entered a plateau at 4–6 h and remained at a relatively stable and high level (Fig. 6B). The expression of IL-17A mRNA in liver tissue showed a similar trend. IL-17A mRNA expression in liver tissues was upregulated during the ischemic phase and significantly upregulated 1–6 h after ischemia (Fig. 6C). It is suggested that IL-17A expression can be induced by both ischemic and reperfusion phases, and a significant increase in the level can be seen at the early stage of reperfusion.

### Experimental validation of the role of the IL-17 pathway and the potential genes in HIRI

**Figure 7:**
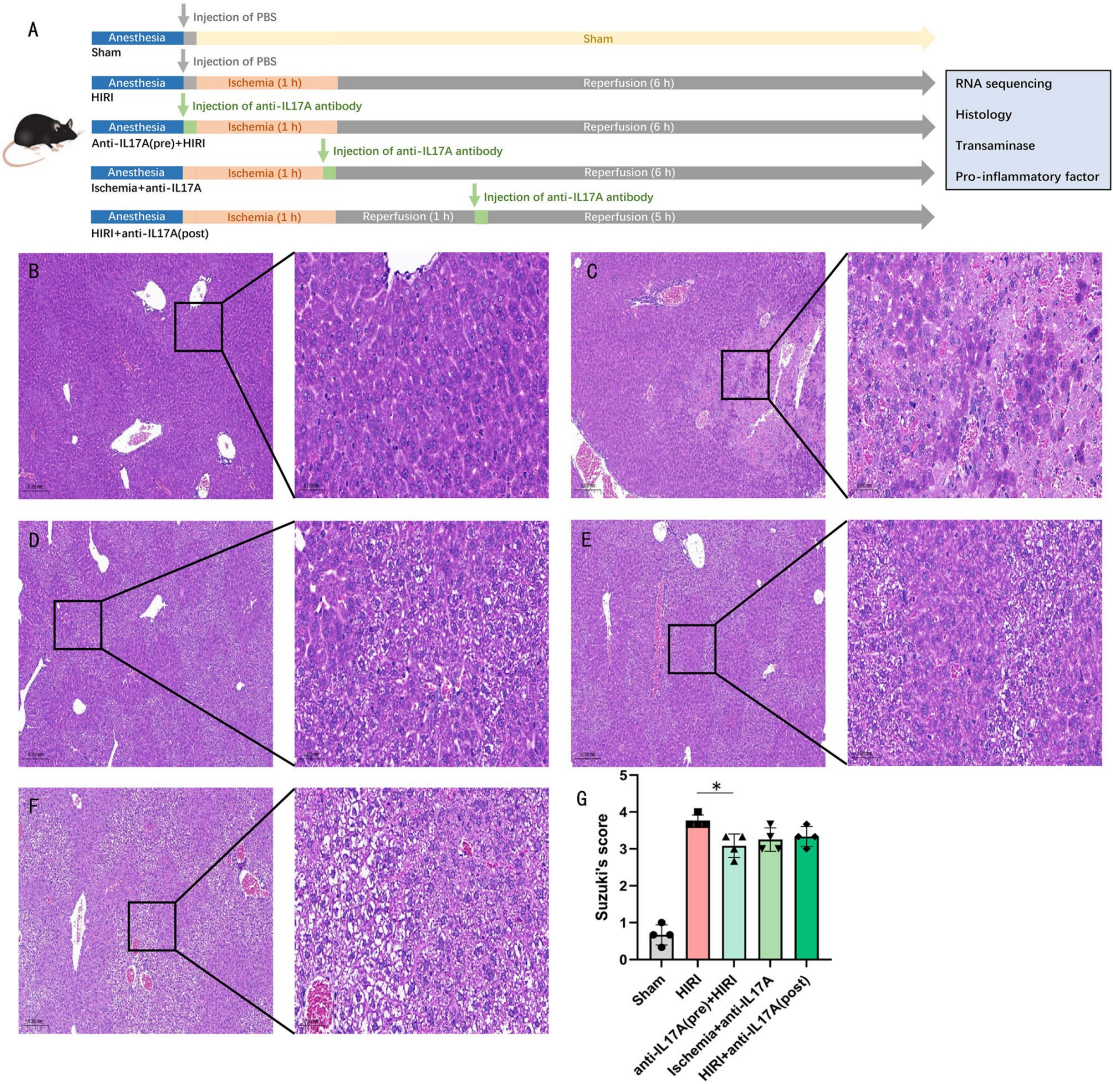
Inhibition of IL-17A ameliorates liver tissue damage after HIRI. (**A**) Pattern diagram of the experiment. (**B**–**F**) Representative image of H&E staining of liver tissues in the sham group (**B**), HIRI group (**C**), anti-IL-17A (pre) + HIRI group (**D**), ischemia + anti-IL17A group (**E**), and HIRI + anti-IL17A (post) group (**F**). (**G**) Suzuki’s quantitative score of the groups. Data results are expressed as mean ± SD (n = 4, each group). *P < 0.05 and **P < 0.01. All data are representative of 3 replicate experiments.

The timing of the intervention and model construction is illustrated in Fig. 7A. After clamping the portal vein branches, arteries, and bile ducts supplying the middle and left lobes of the liver with vascular clips, grayish-white changes due to ischemia were immediately visible to the naked eye in the left and middle lobes of the liver, while the right lobe of the liver appeared to have a normal pinkish color because the blood flow to the liver had not been blocked (SFig. 3A). The livers were isolated after 1 h of ischemia and 6 h of reperfusion, and the diaphragmatic and ventral surfaces of the livers of mice in the Sham group were normal and uniformly pink (SFig. 3B,C), while the middle and left lobes of the livers in the HIRI group showed an abnormal grayish-white color due to HIRI, accompanied by inhomogeneous splotchy alterations (SFig. 3D,E), suggesting that the HIRI model was successfully constructed. Light microscopy showed that in the Sham group, the structure of the liver lobules was intact and clear, with no obvious inflammatory cell infiltration in the confluent area, normal hepatic sinusoids and hepatic cell cord structure, and no obvious hepatocellular degeneration and necrosis (Fig. 7B); in the HIRI group, there was extensive hemorrhagic necrosis of the liver tissues, with a large number of inflammatory cells infiltrated in the confluent area and hepatic sinusoids, and destruction of the structure of hepatic cell cords (Fig. 7C); in the Anti-IL17A (pre) + HIRI group, edematous degeneration, necrosis and scattered vacuo-like degeneration of hepatocytes were observed, with a small infiltration of inflammatory cells in the hepatic sinusoids and the confluent area, and partial destruction of the hepatocyte cord structure (Fig. 7D). Significant liver cell degeneration and structural damage were seen in the Ischemia + anti-IL17A group and the HIRI + anti-IL17A (post) group (Fig. 7E,F). Suzuki's score for liver injury was significantly higher in the HIRI group than in the sham group (P < 0.01), and anti-IL17A antibody pre-intervention improved the liver injury score (P < 0.05) (Fig. 7G). Antibody interventions after ischemia and at 1 h of reperfusion were not effective in remediating liver tissue scores, and a comparison of the effects of the three interventions on liver injury scores did not yield a statistical difference (Fig. 7G).

**Figure 8:**
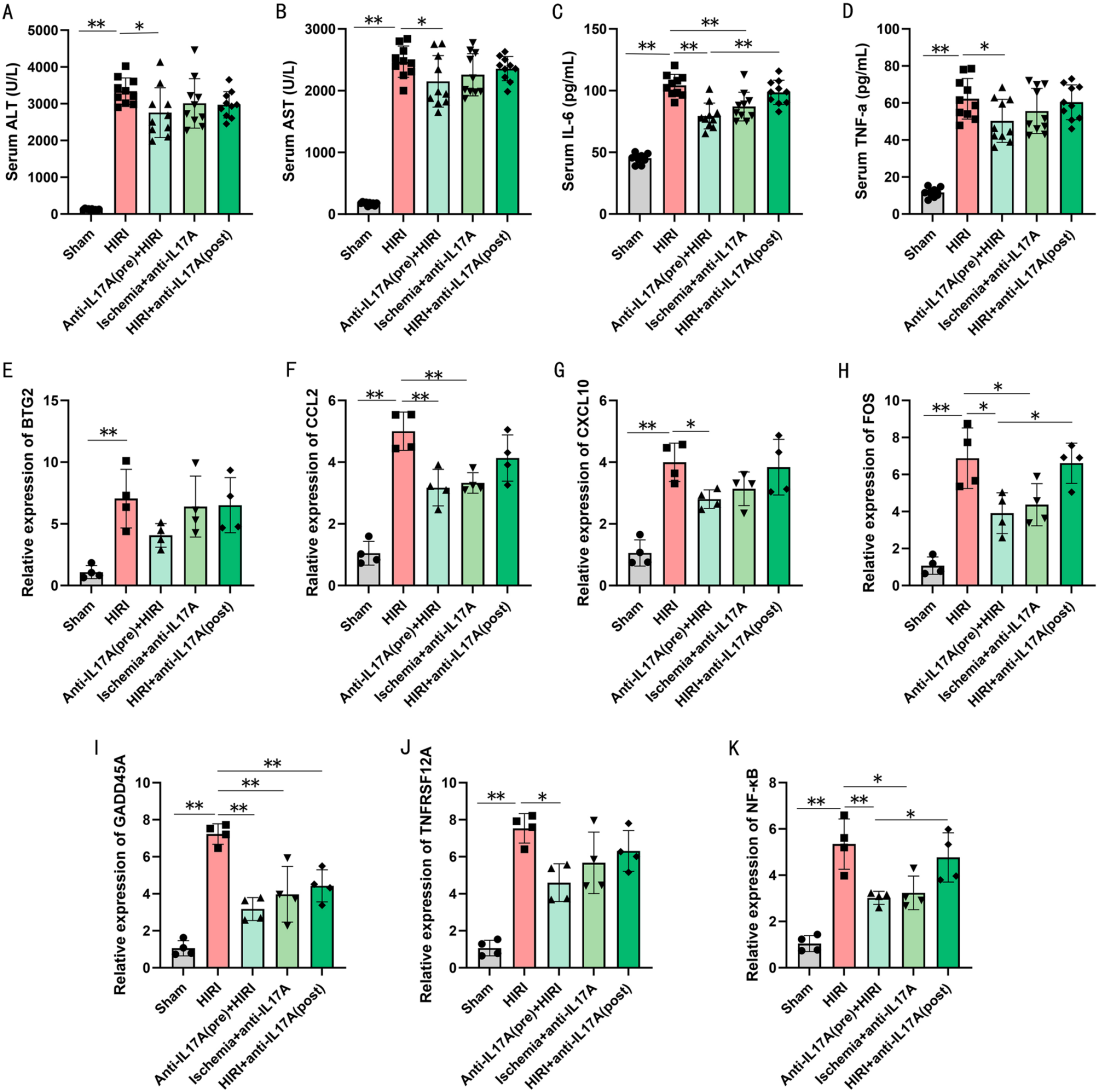
Inhibition of IL-17A reduces the expression of the potential gene and inflammatory response. (**A**–**D**) Serum level of ALT (**A**), AST (**B**), IL-6 (**C**), and TNF-α (**D**), (n = 10, each group). (**E**–**K**) Relative mRNA expression of BTG2 (**E**), CCL2 (**F**), CXCL10 (**G**), FOS (**H**), GADD45A (**I**), TNFRSF12A (**J**), and NF-κB (**K**), (n = 4, each group). Data results are expressed as mean ± SD. *P < 0.05 and **P < 0.01. All data are representative of 3 replicate experiments.

Serum levels of ALT, AST, IL-6, and TNF-α were significantly elevated in the HIRI group compared with the sham group, and preoperative antibody intervention significantly ameliorated serum levels of aminotransferases and inflammatory factors in mice (Fig. 8A–D). Only IL-6 showed a reduction with post-ischemic antibody intervention (Fig. 8C), and the strategy of antibody remediation at 1 h of reperfusion did not show benefits. Expression levels of 6 potential genes (CCL2, FOS, GADD45A, TNFRSF12A, CXCL10, and BTG2), and NF-κB were significantly upregulated after HIRI (Fig. 8E–K). Anti-IL-17A antibody pre-treatment downregulated 5 other potential genes except BTG2 (Fig. 8E) as well as downregulated NF-κB expression levels in HIRI liver tissues. Antibody intervention after ischemia reduced tissue levels of CCL2, FOS, GADD45A, and NF-κB mRNA, of which GADD45A could be similarly downregulated in expression by antibody intervention after reperfusion (Fig. 8I). The above results demonstrated that pre-inhibition of the IL-17 pathway could attenuate HIRI and down-regulate the expression of these genes in mice, and the high expression characteristics of these genes in HIRI model animals also suggested the possibility of their involvement in the core mechanism of HIRI as key molecules, and targeting these genes or proteins to intervene in HIRI may play a beneficial role.

## Discussion

In this study, CCL2, BTG2, GADD45A, FOS, CXCL10, and TNFRSF12A were identified as potential molecules involved in the pathophysiology of HIRI. In the externally validated dataset, except for BTG2 and CXCL10, the remaining four genes showed significant expression differences and good diagnostic efficacy in HIRI. Notably, GSEA functional enrichment of HIRI and non-HIRI specimens showed that the IL-17 pathway was significantly up-regulated in HIRI, and the disease signature genes screened by LASSO, RF, and SVM-RFE algorithms were also significantly enriched in the IL-17 pathway, including a trend that was also reflected in the results of KEGG analyses of the hub module genes by WGCNA, suggesting that IL-17 might be an important pathway involved in pathophysiological processes of HIRI.

In the preliminary phase of this study, we attempted to construct a mouse model of HIRI with partial blood flow blockade for 30 min. However, this method of construction did not yield a statistical difference in SUZUKI's score compared to the livers of mice in the sham group, which made it difficult for us to compare the morphological alterations of the livers under the conditions of antibody intervention (SFig. 4). Therefore, we chose to prolong the hepatic portal blockade to 1 h, thus observing significant liver tissue damage.

We found that IL-17A expression was significantly elevated in the liver and the blood during the ischemic phase of HIRI. Although we intervened with anti-IL-17A antibodies at different HIRI stages in an attempt to counteract the pro-inflammatory effects of IL-17A, interventions at the end of ischemia and 1 h of reperfusion were not as effective as earlier (pre-ischemic) strategies. This phenomenon is interesting and may indicate that a more thorough neutralization strategy may exert a more remarkable efficacy. Since inflammatory immune activation due to acute tissue hypoxia can occur in the ischemic phase, during which a series of signaling cascades mediated by expressed IL-17A through the corresponding receptors induces amplification of the inflammatory response, we hypothesized that inhibition of the inflammatory response before the onset of reperfusion injury (e.g. prior to the onset of ischemia and in the phase of ongoing ischemia) may be effective in attenuating the inflammatory storms that occur as a result of subsequent reperfusion. Thus, neutralization of IL-17A at the end of ischemia also exerted some beneficial effects, including inhibition of IL-6 levels in serum and expression of CCL2, GADD45A, FOS, and NF-κB in liver tissue.

**Figure 9:**
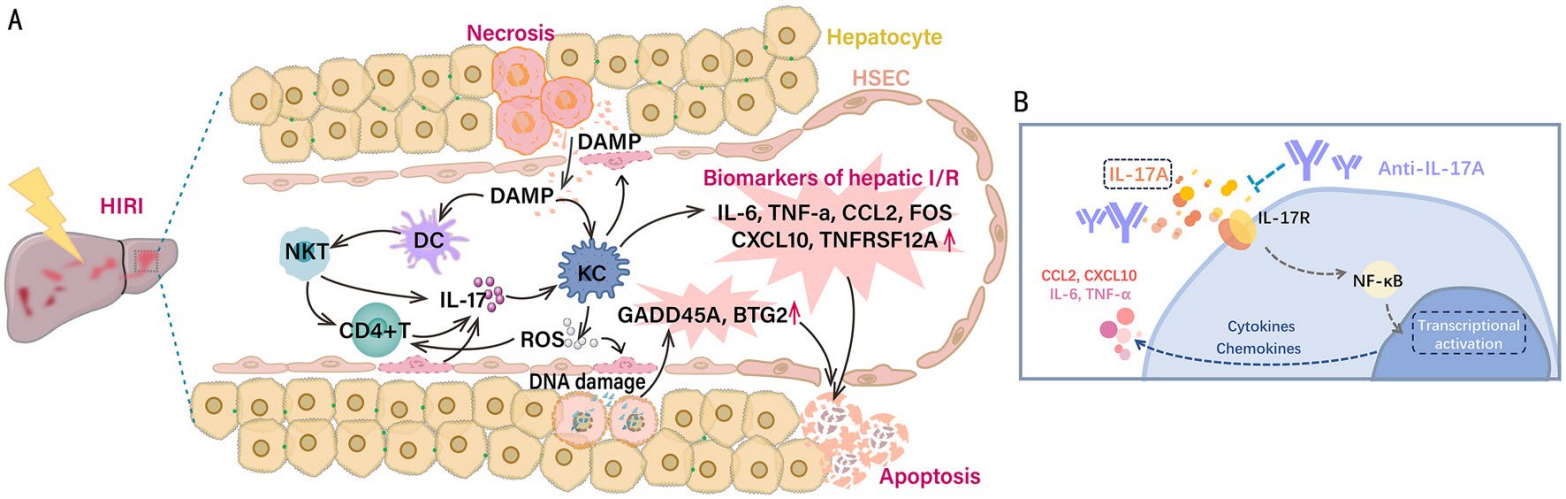
The mechanisms underlying microenvironment remodeling and the IL-17 pathway that are implicated in the pathophysiology of the HIRI. (**A**) The degeneration and necrosis of liver tissue cells during HIRI release a series of endogenous components such as DAMPs, including a variety of nuclear proteins, cytoplasmic proteins, and cytosolic components, which bind to pattern recognition receptors on the membrane surface of immune cells to promote inflammatory immune activation and the downstream pathway to produce a series of pro-inflammatory cytokines and chemokines. Meanwhile, the captured or recognized DAMPs mediate the activation of various immune cells, such as NKT cells and T cells, through the antigen presentation of dendritic cells, and the activated immune cells contribute to the inflammatory signaling cascade through the production and secretion of IL-17 to form an inflammatory storm, leading to the acceleration of the programmed cell death process. In addition, activated macrophages produce ROS, which can further induce the death of liver sinusoidal endothelial cells and other tissue cells, causing upregulation of the expression of DNA damage markers GADD45A and BTG2, creating a vicious cycle. (**B**) Anti-IL-17A antibody inhibits IL-17A binding to the receptor through neutralization. The receptor acts by activation of downstream NF-κB pathway, which ultimately downregulates the expression of disease-related genes, cytokines, and chemokines. *DAMP* damage-associated molecular pattern, *DC* dendritic cell, *NKT* natural killer T cell, *ROS* reactive oxygen species, *KC* Kupffer cells, *HSEC* hepatic sinusoidal endothelial cells, *NF*-*κB* nuclear factor-kappa B.

The specific mechanisms by which the identified genes and IL-17 pathway are involved in the HIRI process remain unknown. The IL-17 family consists of 6 members of ligands (IL-17A ~ IL-17F) and 5 receptors (IL-17RA ~ IL-17RD and SEF). In HIRI, pro-inflammatory signals are emitted by liver resident cells including Kupffer cells and hepatic sinusoidal endothelial cells (HSEC), as well as peripheral immune cells recruited due to chemotaxes, such as monocytes, DCs, T cells, and NK cells. A large number of DAMPs can activate the innate immune system by binding to PRRs on the surface of a variety of immune cells, mediating downstream inflammatory pathways to generate a range of cytokines and chemokines. As a conserved T cell subline, NKT cells can be activated and then rapidly produce cytokines in response to antigen presentation by DCs^19,20^, while CXCL6-expressing DCs can bind to NKTs expressing the chemokine receptor CXCR6 to promote NKT cell activation/maturation^21^. Activated NKT cells promote CD4^+^ T cell differentiation and act on Kupffer cells by further generating IL-17, inducing downstream pro-inflammatory mediator expression^22–25^, and generating large amounts of ROS, which can further act on HSEC and hepatocytes to promote programmed cellular death or necrosis, expanding the inflammatory cascade^26^ (Fig. 9A).

As a member of the Th17 cytokine family, IL-17A is mainly produced by CD4^+^ Th17 cells, innate lymphoid-like cells, and typical innate immune cells such as NK cells and neutrophils^27,28^. IL-17A plays an important role in regulating host autoimmunity, various inflammatory diseases, and tumor immunity. Recent studies have identified its pro-inflammatory role in ischemia–reperfusion injury in the myocardium, intestine, and liver by regulating parenchymal apoptosis and neutrophil infiltration. Activation of IL-17A signaling during inflammatory stress leads to increased reactivity to inflammatory stimuli and recruitment of immune cells to the liver. Accumulating evidence demonstrates the involvement of IL-17A in the pathological process of HIRI, including significantly elevated expression levels correlating with blood transaminase levels, enhanced hepatic necrosis, and more severe immune cell infiltration in HIRI mice^29^. On the one hand, IL-17A binds to the receptor to activate the IL-17 pathway, which leads to downstream nuclear translocation and transcriptional activation of NF-κB, causing the production of various pro-inflammatory mediators such as cytokines and chemokines. On the other hand, in the initial stage of HIRI, IL-6, IL-21, and IL-23 produced in the locally ischemic liver lobes lead to the stimulation of immune cells. The phosphorylation level of the intracellular transcription factor STAT3, which is associated with the production of IL-17A, is increased, which promotes IL-17A production, so that as HIRI progresses, it is accompanied by the continuous accumulation of IL17-related mediators and amplification of inflammatory signaling^27,28^. In signaling downstream of IL-17, Act1 is a unique cytoplasmic adapter required for the activation of all known IL-17-dependent signaling pathways by interacting with IL-17Rs, and contains a TNF receptor-associated factor (TRAF)-binding site that binds to TRAF-family proteins. Binding to TRAF6 drives activation of the NF-κB pathway, which together trigger transcriptional induction of the target gene^30^. We found that NF-κB mRNA expression was significantly upregulated in the liver tissues of HIRI mice and were both suppressed after injection of anti-IL-17A antibody, suggesting that inhibition of the IL-17 pathway and its downstream NF-κB pathway could down-regulate the key genes and multiple cytokine and chemokine expressions associated with HIRI. (Figs. 7K, 9B). Current studies have focused on the roles and mechanisms of IL-17A and its receptors involved in HIRI, and our study provides information on the characterization of IL-17A expression and the timing of intervention in HIRI. However, further studies on the expression landscapes of different IL-17 family members and their corresponding receptors in HIRI and the mechanisms involved are needed.

Synthesizing public data and experimental results, four genes, CCL2, GADD45A, FOS, and TNFRSF12A, were identified as key molecules involved in HIRI. Among them, CCL2, as a chemokine, is a key link in the recruitment and activation of immune cells and subsequent liver injury. In previous studies, DAMPs released from tissue cell death in HIRI promoted transcription and secretion of CCL2 through activation of immune cells expressing PRRs, such as Kupffer cells^31^, thereby recruiting CCR2-expressing bone marrow-derived neutrophils to infiltrate the liver^32^. CCL2 recruits peri-injured monocytes and neutrophils through chemotaxis. Compared to wild-type, mice defective in CCL2 or its receptor, C–C chemokine receptor 2 (CCR2), exhibit reduced accumulation of inflammatory monocytes or neutrophils and attenuated hepatic injury after HIRI^18,32^. In mammals, GADD45A is widely expressed in the liver, kidney, heart, skeletal muscle, etc.^33^. As a stress protein that responds to the environment, GADD45A plays an important role in toxic and non-toxic stress responses, regulating DNA repair, cell cycle, and senescence. Upon DNA damage, GADD45A is rapidly produced in the cell to participate in the DNA damage repair process, and at the same time, it induces cell cycle arrest and/or apoptosis^34^. As HIRI is often accompanied by acute and severe impairment of mitochondrial and DNA damage^35^, given which GADD45A is up-regulated and involved in pathological processes in HIRI, a small number of studies have reported on the predictive and diagnostic potential of GADD45A in drug-induced liver injury^36,37^. However, there are currently very few studies reporting on the exact mechanism of GADD45A involvement in HIRI. FOS, together with CCL2, is at a key position in the protein interaction network. FOS is a member of the FOS family, and the gene encodes a transcription factor that forms a dimer with members of the Jun family. The dimerization complex binds to DNA at specific sites of AP-1 in the promoter and enhancer regions of target genes, thereby translating extracellular signals into changes in gene expression, such as activation of the NF-κB pathway, which is involved in the regulation of inflammatory activation and cell survival-related processes^38^. Several studies have shown that in HIRI, the MAPK-extracellular signal-regulated kinase (ERK) signaling pathway is usually in an abnormally high state of activity, with multiple cytokines activating ERK1 and ERK2^39,40^, and that activated ERK phosphorylates the c-Fos transcription factor, which in turn plays a direct role in inflammatory regulation^41^. Similarly, FOS was identified as a potential risk factor for aging livers to be more susceptible to HIRI in human liver transplantation sequencing profiles, suggesting an important role of FOS involved in the pro-inflammatory effects of HIRI^42^. The exact mechanism by which TNFRSF2A is involved in HIRI is currently unknown. Relevant studies have shown that the expression level of TNFRSF12A is extremely low in normal livers that do not undergo stress events such as inflammation, but the expression of this gene is significantly increased in affected liver tissues of obstructive cholestasis or primary biliary cholangitis^16^. TNFRSF12A expression can be induced by enhancing the binding of the transcription factor c-JUN to the TNFRSF12A promoter and can initiate hepatocyte cellular pyroptosis through downstream NF-κB/Caspase-1/GSDMD signaling^16^. As a classical ligand for TNFRSF12A, tumor necrosis factor-related weak inducer of apoptosis (TWEAK) can be secreted by infiltrating macrophages in the liver, and the expression of TWEAK and TNFRSF12A was significantly increased during paracetamol-induced acute liver failure, and TNFRSF12A-induced apoptosis of hepatocytes could be significantly attenuated by the inhibition of the TWEAK/TNFRSF12A axis^43^. In addition, although none of the CXCL10 expressions in HIRI showed significant differences relative to normal liver samples in external validation, CXCL10 expression was significantly increased after HIRI in animal experiments (P < 0.01) and decreased after anti-IL17 antibody intervention (P < 0.05). It was found that CXCL10 expression could be significantly increased 1 h after the occurrence of HIRI, and CXCL10 knockout mice were observed to have reduced serum ALT levels, attenuated expression of pro-inflammatory genes such as tissue TNF-α, IL-1β, IL-12β, and alleviated the damage of liver tissue after HIRI^44^. Hepatic sinusoidal endothelial cells in the affected liver inhibit NK cell infiltration in tissues after CXCL10 knockout in a sepsis model^45^. BTG2 has been found to be associated with modulating tumor cytological behavior and regulating cell differentiation in previous studies^46,47^. Genotoxicity or DNA damage can induce upregulation of BTG2 expression, which is involved in DNA damage repair or induction of apoptosis^48^. In the present study, anti-IL-17A antibody intervention failed to reduce BTG2 mRNA expression in liver tissues of HIRI mice, suggesting that BTG2 may be involved in the process of HIRI through a signaling mechanism independent of IL-7. Interestingly, BTG2 expression was recently found to be significantly elevated in liver tissues of mice or humans with alcoholic liver disease, while elevated expression of this gene was also seen in liver tissues of fasted and diabetic mice and diabetic patients, where overexpression of BTG2 enhances cyclic adenosine monophosphate (cAMP)-responsive element-binding protein H to promote hepatic gluconeogenesis^49,50^. Considering that the construction of HIRI model usually requires mice to be fasted for 6 h, whether the alteration of BTG2 expression level due to fasting will affect the process of HIRI needs further investigation.

Considering that HIRI is a pathological process centered on aseptic inflammation, after functional enrichment of the WGCNA red module, we demonstrated the top five ranked biological pathways, mainly associated with pro-inflammatory responses. We found that in addition to the core genes identified, RELA, CCL2, and CCL7 were significantly enriched in the IL17 pathway and the TNF pathway (P < 0.001, Fig. 3D, Supplemental Table 3). RELA (p65), a member of the NF-κB family of transcription factors, is involved in key processes of inflammatory regulation in mammals. The inflammatory cascade involved in RELA was demonstrated in recent studies, where STAT3 could promote IGF2BP1-RELA-mediated pro-inflammatory effects by binding to the insulin-like growth factor 2 mRNA-binding protein 1 (IGF2BP1) promoter^51^. Inhibition of RELA transcription attenuates liver and lung injury in septic mice^52,53^. In cardiomyocytes, activation of adenylate cyclase type 8 leads to cell-autonomous RELA-mediated NF-κB signaling, which induces a widespread and persistent inflammatory state^54^. At HIRI, the ease of p65 nuclear translocation can enhance cGAS transcription by binding to the promoter of cGAS and further via the STING pathway thereby exacerbating hepatocyte injury^55^. In addition to CCL2, which we identified as a key gene, several studies have reported that CCL7 is involved in the process of acute liver injury induced by LPS or chronic liver injury constructed by methionine- and choline-deficient dietary feeding and that hepatotoxicity induced by acetaminophen in mice can be enhanced by overexpression of CCL7^56,57^. Although all of these studies suggest an important role for these genes in the inflammatory immune process, the specific roles and mechanisms of these genes involved in HIRI are unclear and require further investigation.

There are some limitations to this study. Firstly, we focused only on transcriptome alterations before and after the onset of HIRI in both sequencing datasets, without identifying changes in expression dynamics at at different stages of HIRI. Secondly, although we analyzed 20 normal and 19 HIRI samples from two microarray datasets, the sample sizes are still small. Due to the difficulty of obtaining human liver samples after hepatectomy or liver transplantation, we did not validate the expression and diagnostic efficacy of the screened key genes on liver tissues of surgical patients after the occurrence of HIRI, and we did not clinically investigate the efficacy and feasibility of the intervention of anti-IL-17A antibody in patients with HIRI, which are to be investigated in the next step.

## Conclusion

In conclusion, this study combined bioinformatics technology and animal experiments to screen and validate the core molecules and pathways involved in HIRI and investigated the effects of anti-IL-17A antibodies on attenuating HIRI. The results suggest that CCL2, FOS, GADD45A, and TNFRSF12A are the key genes involved in the development of HIRI, and that pre-administration of anti-IL-17A antibodies can effectively down-regulate the expression of the above genes, inhibit inflammatory response and attenuate HIRI.

### Supplementary Information

Supplementary Figures.

Supplementary Tables.

## Data Availability

The datasets used and/or analysed during the current study available from the corresponding author on reasonable request.
