# Supplementary material for: Analysis and experimental validation of IL-17 pathway and key genes as central roles associated with inflammation in hepatic ischemia–reperfusion injury
Source: Sci Rep. 2024 Mar 18;14:6423. doi: 10.1038/s41598-024-57139-2 (PMC10944831; doi:10.1038/s41598-024-57139-2)
Supplement: Supplementary file 1 — Supplementary Figures. [file 41598_2024_57139_MOESM1_ESM.docx]

**Supplementary materials**

**Figures**

**
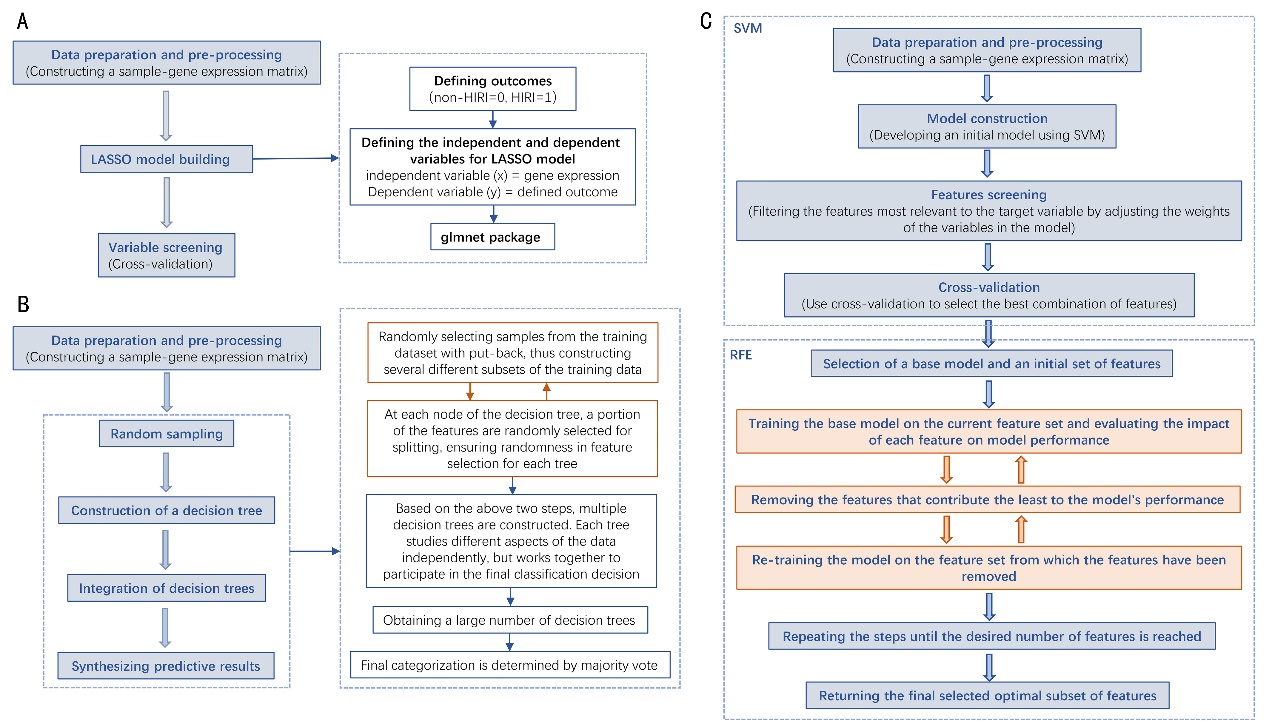
**

**SFigure 1. Operation flow of three machine learning algorithms** (A) Operation flow of LASSO algorithm. (B) Operation flow of Random Forest. (C) The operational flow of the SVM-RFE algorithm.

**
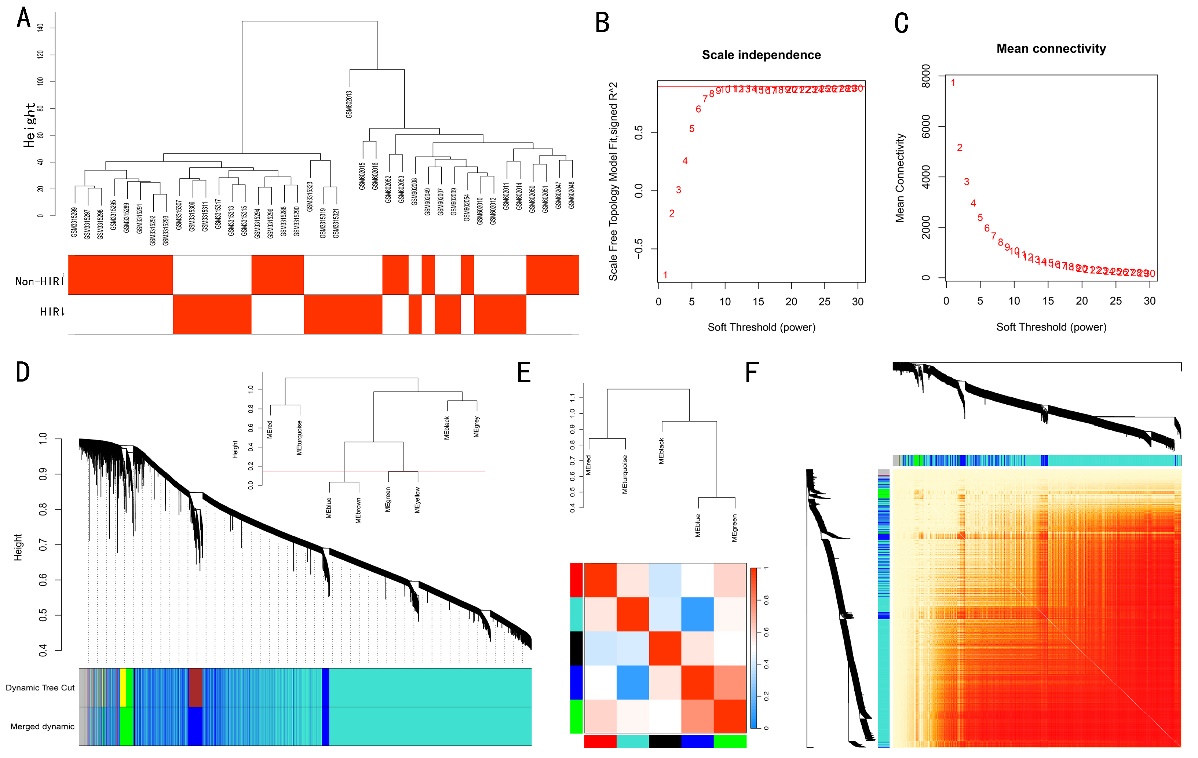
**

**SFigure 2. Weighted gene co-expression network analysis.** (A) Sample clustering dendrogram with tree leaves corresponding to individual samples. (B, C) Analysis of the scale-free fit index (R2) and the mean connectivity for various soft-thresholding powers. (D) The original and combined modules under the clustering tree. (E) Collinear heat map of module feature genes. Red indicates a high correlation, and blue indicates the opposite results. (F) Clustering dendrogram of module feature genes.

**
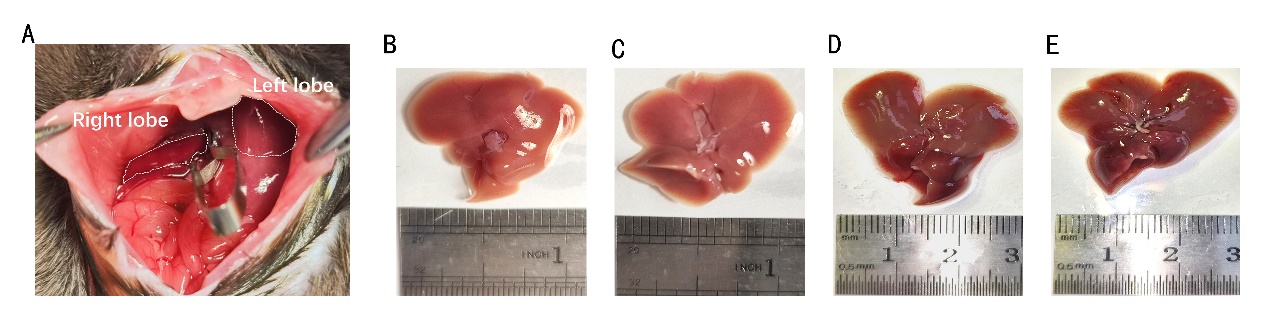
**

**SFigure 3. Naked eye view of 70% HIRI model construction.** (A) Photo of the liver under partial portal blockade. (B-C) Diaphragmatic (B) and ventral (C) views of the liver in sham group mice. (D-E) Diaphragmatic (D) and ventral (E) views of the liver in HIRI group mice.

**
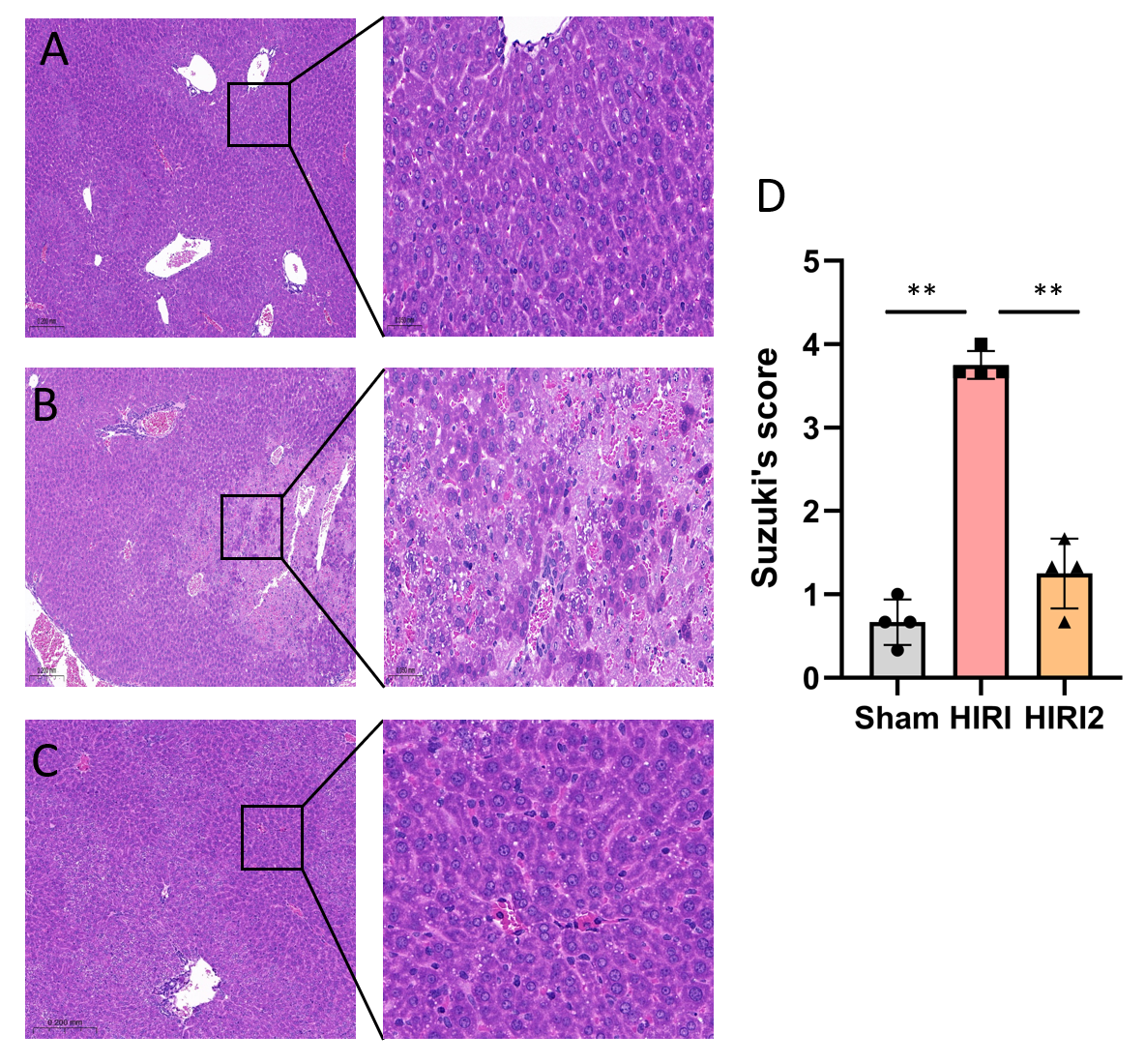
**

**SFigure 4. Liver histomorphometry and damage scoring.** (A-C) Representative image of H&E staining of liver tissues in the sham group (A), HIRI (1 h of ischemia and 6 h of reperfusion) group (B), and HIRI2 (30 min of ischemia and 6 h of reperfusion) group (C). (D) Suzuki’s quantitative score of the groups. Data results are expressed as mean ± SD (n = 4, each group). **P < 0.01. All data are representative of 3 replicate experiments.
